# Morel-Lavallée Lesion of the Knee in a Recreational Frisbee Player

**DOI:** 10.1155/2016/8723489

**Published:** 2016-07-14

**Authors:** Alison Shmerling, Jonathan T. Bravman, Morteza Khodaee

**Affiliations:** ^1^Department of Family Medicine, University of Colorado School of Medicine, Denver, CO 80238, USA; ^2^CU Sports Medicine, Division of Sports Medicine and Shoulder Surgery, Department of Orthopaedics, University of Colorado School of Medicine, Denver, CO 80238, USA; ^3^Department of Family Medicine, AFW Clinic, University of Colorado School of Medicine, 3055 Roslyn Street, Denver, CO 80238, USA

## Abstract

Traumatic swelling/effusion in the knee region is a relatively common presenting complaint among athletes and nonathletes. Due to its broad differential diagnosis, a comprehensive evaluation beginning with history and physical examination are recommended. Knee joint effusion can be differentiated from other types of swelling by careful physical examination. Imaging, including plain radiography, ultrasound, and magnetic resonance imaging (MRI), is preferred modality. Aspiration of a local fluctuating mass may help with the diagnosis and management of some of these conditions. We present a case of a 26-year-old gentleman with superomedial Morel-Lavallée lesion (MLL) of the knee with history of a fall during a Frisbee game. His MLL was successfully treated with therapeutic aspiration and compression wrap without further sequelae. MLL is a rare condition consisting of a closed degloving injury caused by pressure and shear stress between the subcutaneous tissue and the superficial fascia or bone. Most commonly, MLL is found over the greater trochanter and sacrum but in rare cases can occur in other regions of the body. In most cases, concurrent severe injury mechanisms and concomitant fractures are present. MLL due to sports injuries are very rare. Therapeutic strategies may vary from compression wraps and aspiration to surgical evacuation.

## 1. Introduction

Effusions and swelling in the knee region are common presenting complaints among athletes and nonathletes. With a thorough history and physical examination, particularly with a history of trauma, infectious and inflammatory causes can often be ruled out. The time course of a traumatic knee effusion is also important to incorporate, as an effusion evolving within four hours of injury increases the likelihood of major osseous, ligamentous, or meniscal injury [1]. Morel-Lavallée lesions (MLL) is a rare condition presenting with superficial fluid collection between subcutaneous tissue and the superficial fascia or bone mainly caused by direct trauma. MLLs are a structural cause of knee swelling which are often missed or late diagnosed, in part because their occurrence at the knee is only more recently appreciated [2]. With MLL, the lesion can present anywhere from a few hours after the injury or as late as 13 years later, making the diagnosis more challenging [3]. Fortunately, with imaging techniques such as ultrasound and MRI, and procedures such as aspiration, MLL is increasingly diagnosed as the etiology of traumatic periarticular knee swelling. This case describes an uncharacteristic MLL found in the knee of a recreational Frisbee player. There have been only few case reports of sports related knee MLL.

## 2. Case Report

**Figure 1:**
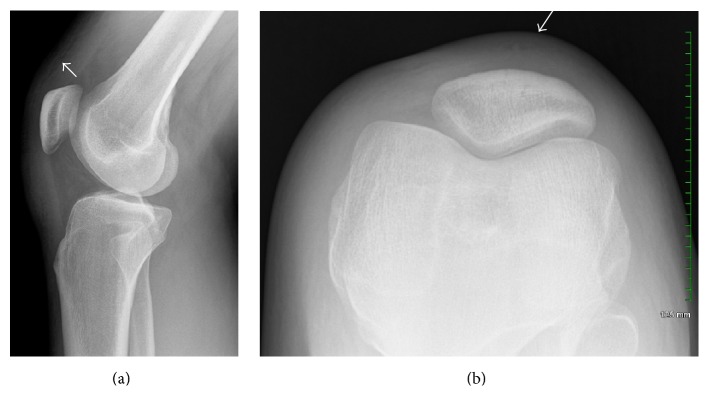
Plain radiography of the right knee. Lateral (a) and sunrise (b) views revealed anterior soft tissue swelling particularly in the superomedial patellar region (arrows).

**Figure 2:**
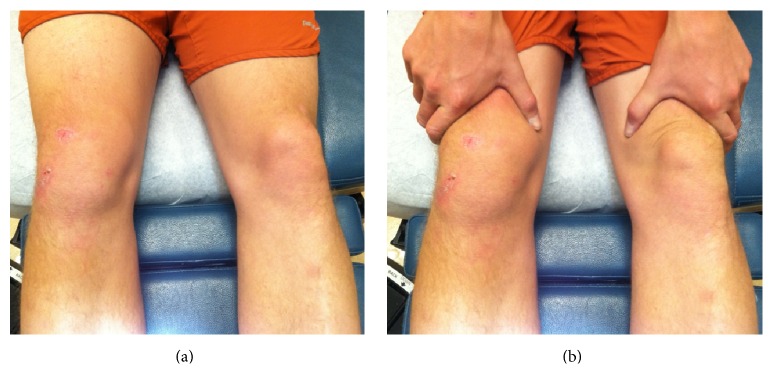
Moderate swelling/effusion in the superomedial aspect of right knee (a) which is accentuated by milking the suprapatellar tissue inferiorly (b).

**Figure 3:**
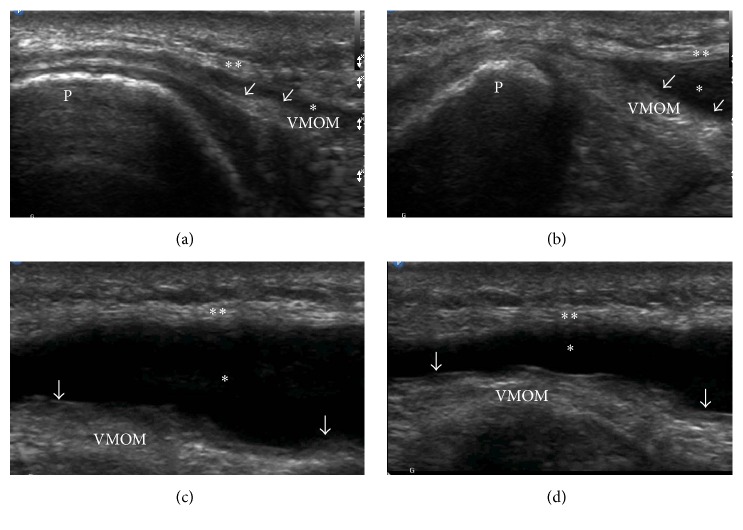
Using a linear transducer (Philips L12–3 MHz) an area of homogenous anechoic fluid collection with scattered hyperechoic substance (*∗*) between subcutaneous tissue (*∗∗*) and superficial quadriceps fascia (arrows) was visualized. Long-axis middle suprapatellar view (a), long-axis medial suprapatellar view (b), short-axis medial suprapatellar view (c), and compressible fluid collection in short-axis suprapatellar view (d). Patellae (P) and vastus medialis oblique muscle (VMOM) look unremarkable with no signs of prepatellar bursal enlargement.

**Figure 4:**
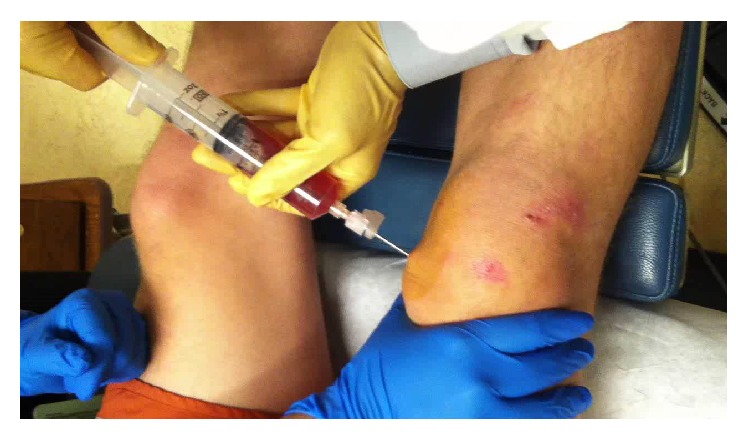
Using ultrasound for needle placement, 38 mL serosanguinous fluid was aspirated.

A 26-year-old gentleman presented to his primary care physician with right knee swelling after a direct fall on his knee during a Frisbee game 2 days earlier. He denied hearing or feeling a popping sensation. The swelling had developed over a few hours but started diminishing since the day before the visit. His moderate pain has been improving since the incident. He had been able to walk with minimum discomfort. His past medical, social, and family histories were unremarkable. On physical examination, he had a mild ecchymosis and abrasion in the superior aspect of his knee with mild swelling. Plain radiography demonstrated soft tissue swelling anteriorly without osseous abnormality (Figure 1). He was advised to rest and use ibuprofen as needed for pain. He presented to our sports medicine clinic with continuous, painless swelling in the same region 19 days after injury. He denied mechanical symptoms and his physical examination was significant for a nontender, moderate sized swelling in the superomedial aspect of his right knee (Figure 2). There was no palpable joint effusion. In-office ultrasound revealed a homogeneous, anechoic fluid collection with scattered hyperechoic substance between the superficial quadriceps fascia and subcutaneous tissue which was compressible (Figure 3). After proper cleansing and local anesthesia with 1% lidocaine, using ultrasound for needle placement and an 18-gauge needle, 38 mL serosanguinous fluid was aspirated (Figure 4). Patient was advised to use a compression wrap following the procedure. He presented for recurrence of his knee swelling on day 25 after injury. Another aspiration provided 35 mL of serosanguinous fluid. After the second aspiration, his symptoms were completely resolved with no reaccumulation of the fluid. At latest follow-up, 4 weeks from injury, he is asymptomatic and had returned to full, unrestricted activity.

## 3. Discussion

MLL is a rare condition consisting of a closed degloving injury caused by tangential impact and shear stress between the subcutaneous tissue and the muscle fascia or bone [4]. The potential space between these tissues is subsequently filled with serous, blood, lymphatic fluid, or necrotic fat [5, 6]. Most commonly, this lesion is found over the greater trochanter but can be found in other regions of the body [5–7]. Classic history includes crush injury, with soft fluctuant area appreciable on physical examination [3, 5]. MLL has been rarely reported in the knee region [2, 8–12] and as a result of sports injuries [10, 13–16]. In some chronic cases, the history of a significant trauma may not be present [16]. For these reasons, MLLs are often misdiagnosed. The natural course is not well understood, with the lesion potentially enlarging in size, remaining stable, or self-resolving. This depends on the content of the fluid and stages of hematoma formation [5, 17, 18]. In some cases, it may recur [5, 18].

Diagnosis can be made clinically. Ultrasound may reveal hypoechoic or anechoic collection which is typically compressible and usually located between deep fat and overlying fascia, regardless of age of the lesion [4]. Lesions <1 month old appear heterogeneous with irregular margins and lobular shape, while lesions >18 months old tend to appear more homogenous and have a flat or fusiform shape with smooth margins [4, 17]. MRI can also be used to diagnose MLL [4, 5, 11, 12, 17] and can help classify MLL into different types based on T1 and T2 characteristics of the lesions [5]. Mellado and Bencardino classified the MLL into six types [18]. Type I is a serohematic effusion, type II is a subacute hematoma, type III is a chronic organizing hematoma, type IV is perifascial dissection with closed fatty laceration, type V is a perifascial pseudonodular lesion, and type VI characterizes as an infected lesion with multiple sinus tract formation, internal septations, and thick capsule [5, 18]. With MRI, the age of the lesion is more easily appreciated [5].

Treatment varies from watchful waiting to drainage and compression/pressure, with surgical intervention as a last resort [3, 5, 19]. Percutaneous aspiration with a large-bore needle (14–22 gauges) is recommended, particularly being performed with ultrasound guidance, both to aid with diagnosis and to treat the lesion. Depending on the stage of hematoma formation, aspiration may not provide any fluid. Immediate compression after aspiration may help prevent reaccumulation [10, 11]. Sclerosing agents such as doxycycline, erythromycin, alcohol, bleomycin, or talc can be used on chronic lesions [5] and surgery may be performed for refractory cases, which may involve excision of the pseudocapsule and necrotic tissue debridement [3, 5, 19]. The wound is then either left open, placed to vacuum seal, or closed with or without a drain [11].

Based on few case reports including this case, it seems that MLLs as a result of low energy and sports injuries typically have a favorable outcome with full return to physical activities and no further sequelae.
